# Cascading Robustness Analysis of Wireless Sensor Networks with Varying Multisink Placement

**DOI:** 10.3390/s23115337

**Published:** 2023-06-05

**Authors:** Lin Ding, Dan Sheng, Minsheng Tan, Juan Wen

**Affiliations:** 1 School of Computer, University of South China, Hengyang 421001, China; 2 School of Electrical Engineering, University of South China, Hengyang 421001, China

**Keywords:** wireless sensor networks, cascading failures, multisink placement, routing scheme, robustness

## Abstract

In practical wireless sensor networks (WSNs), cascading failures are closely related to network load distribution, which in turn strongly relies on the locations of multiple sink nodes. For such a network, understanding how the multisink placement affects its cascading robustness is essential but still largely missing in the field of complex networks. To this end, this paper puts forward an actual cascading model for WSNs based on the multisink-oriented load distribution characteristics, in which two load redistribution mechanisms (i.e., global routing and local routing) are designed to imitate the most commonly used routing schemes. On this basis, a number of topological parameters are considered to quantify the sinks’ locations, and then, the relationship between these quantities with network robustness is investigated on two typical WSN topologies. Moreover, by employing the simulated annealing approach, we find the optimal multisink placement for maximizing network robustness and compare the topological quantities before and after the optimization to validate our findings. The results indicate that for the sake of enhancing the cascading robustness of a WSN, it is better to place its sinks as hubs and decentralize these sinks, which is independent of network structure and routing scheme.

## 1. Introduction

As an essential part of the Internet of Things (IoT) system, wireless sensor networks (WSNs) are increasingly widely used in various fields, such as military, industry, transportation and environmental protection. In general, WSNs are composed of numerous low-cost sensors with data processing and wireless communication capabilities, which automatically collect environmental data in a self-organized manner and forward them to sinks through one-hop or multi-hop routing [1,2,3]. However, in real-life applications, these nodes often fail due to unpredictable events, such as energy depletion, hardware malfunction, or deliberate attacks. When a sensor fails, the data originally passing through the faulty node have to be rerouted. This rerouting process of redistribution of load may lead to overload failures on more nodes and further cause a new round of cascading failures [4,5]. Consequently, the whole network is largely affected or even globally collapsed. To avoid or at least reduce such catastrophic failures, enhancing the robustness of WSNs has been a research hotspot in recent years.

Complex network theory is now a powerful tool used in the research on the robustness of realistic network systems [6,7]. By abstracting WSNs into graphs, researchers have proposed various models to describe the cascading failure process [8,9,10,11,12,13]. Based on these models, it has been widely proved that the cascading performance of WSNs largely depends on the underlying network topology and the routing scheme implemented. So, current methods to enhance network robustness can be mainly categorized into two groups. One is modifying network topological structure [14,15,16] and the other is designing a better routing scheme [17,18,19].

In most of these existing studies, the cascading models for WSNs are developed based on general topological load metrics (i.e., degree or betweenness). The premise of those models is that the data transmission is conducted in a peer-to-peer mode, that is, data can be transferred between any pair of network nodes. However, in reality, all data in WSNs will be transmitted from general sensors to sinks, and the data transmission mode oriented to multiple sinks has obvious advantages in energy efficiency and reliability compared with only one sink [20,21,22]. This makes real-life WSNs exhibit different load distribution characteristics from the traditional peer-to-peer networks. In this kind of multisink network, the placement of sinks falls under the topological planning. It would be expected that given a sensor-to-sink routing scheme, if the sinks’ locations in the network topology are changed, the data will arrive at the destinations along the different paths, and the traffic load will change accordingly. Therefore, the multisink placement is an important factor affecting the network load distribution, which may further affect the network cascading process. Unfortunately, although there are some recent studies on multisink placement in a WSN, hardly any attention is paid to quantifying network robustness to cascading failures in terms of the topological location properties of its sinks.

Specifically, current studies on the multisink placement mainly focus on various optimization approaches that only aim to find the optimal locations of sinks to maximize network performance in terms of transmission latency, network lifetime, or other aspects [23,24,25,26], while much less effort has been made in terms of network cascading robustness. The particle swarm algorithm [27], genetic algorithm [28], and memetic algorithm [29] are some of efficient tools adopted to solve such optimization problems. It needs to be noticed that these tools can present the optimal results for specific problems studied, but they can hardly give any insight into how the multisink placement may affect the network performance. Therefore, so far, there is a lack of understanding of the relationship between topological location properties of sinks and cascading failures for a given WSN.

In this paper, we develop a systematic study to fill this gap. We first construct an actual cascading model for multisink WSNs. Based on this model, a number of topological parameters are introduced to establish the connectivity between cascading failures and multisink placement. The new findings obtained here reveal the impact of varying topological location properties of sinks and its association with the network configuration considering network structure and routing scheme, providing insight into optimizing or designing WSNs to make them more robust against cascading failures. The main contributions of this paper are summarized as follows:

(1) We put forward a multisink-oriented cascading model for WSNs to characterize the actual cascading process of WSNs, which considers the multisink-oriented load distribution characteristics and the two most commonly used routing schemes.
(2) We introduce five topological parameters to give quantitative measures of the sinks’ locations for WSNs.
(3) We carry out an experimental analysis on the relationship between the topological location parameters of sinks and network robustness on the two typical network topologies imitating real-life WSN conditions.
(4) We design a simulated annealing algorithm to maximize network robustness through optimizing the multisink placement and compare the topological quantities before and after optimization to validate the experimental results.

The rest of the article is arranged as follows. Section 2 details the constructed multisink-oriented cascading model for WSNs. Section 3 discusses the impacts of topological parameters for multisink placement on robustness. Section 4 introduces a simulated annealing algorithm to find the optimal multisink placement and compares the topological quantities before and after optimization to validate our findings. Section 5 concludes this article.

## 2. Multisink-Oriented Cascading Model for WSNs

In this section, we elaborate the constructed cascading model for WSNs in detail. First, we present the network description and introduce a load metric called multisink-oriented betweenness to capture the load distribution of WSNs. On this basis, we give the definition of the initial load and capacity of sensors. Then, to describe the process of multisink-oriented load redistribution in common routing schemes, we propose two load redistribution schemes. Finally, we discuss the cascading mechanism and robustness measure.

### 2.1. Network Description

In this study, a WSN can be abstracted by an undirected graph G=(V,E), where $V=V_{G}\cup V_{S}$ is the node set consisting of general sensors and sinks. $V_{G}=\left\{1,2,\cdot \cdot \cdot ,N_{G}\right\}$ and $V_{S}=\left\{1,2,\cdot \cdot \cdot ,N_{S}\right\}$ are the sets of general sensors and sinks, respectively. $N=N_{G}+N_{S}$ is the total number of nodes in the network. The N×N adjacency matrix $\left[a_{ij}\right]$ has $a_{ij}=1$ if node *i* is connected with node *j*; otherwise, $a_{ij}=\;0$. For actual WSNs, a realistic situation is that no direct links exist between sinks, so the link set *E* does not contain the link created within the set of sinks.

### 2.2. Initial Load and Capacity

In an actual WSN, general sensors collect data or act as routers for transmitting data to sinks. Usually, the data flow from the sensors to their nearest sinks through the shortest path and maintain a balanced state owing to a long time evolving; thus, the initial load of a sensor relates to the number of shortest paths from all sensors to their nearest sinks that pass it in the network [16]. To properly reflect such multisink-oriented load distribution characteristics, different from the widely used general betweenness-load metrics based on the shortest path of any node pair, we define a metric called multisink-oriented betweenness (MB) to describe the initial load on sensors, i.e.,

(1) $$\begin{array}{c} MB_{i}=\sum_{j\in V_{G}}\sum_{m\in S(j)}\frac{\omega_{j,m,i}/\omega_{j,m}}{N_{G}N_{S(j)}}, \end{array}$$

where S(j) represents the set of sinks that are closest to sensor *j* and $\omega_{j,m,i}$ is the number of the shortest paths going from sensor *j* to its nearest sink *m* that run through sensor *i*. $\omega_{j,m}$ denotes the total number of available shortest paths going from sensor *j* to its nearest sink *m*. $N_{S(j)}$ is the size of the sink set S(j). Then, the initial load of a sensor *i*, denoted by $L_{i}\left(0\right)$, can be estimated by its multisink-oriented betweenness $MB_{i}$, i.e., $L_{i}\left(0\right)=MB_{i}$.

In actual WSNs, the capacity of a sensor, which characterizes the maximum load this node can manipulate, is limited by its buffer size. The existing cascading models usually assume that the capacity is directly related to the initial load of the node [10,13]. However, in most real-life cases, WSNs cannot customize the capacity of sensors according to their initial load since the hardware configuration of sensors in the same network is identical under the unified large-scale deployment [30]. In this way, the capacity of each sensor can be defined as

(2) $$\begin{array}{c} W=\left(1+\beta \right)\frac{\sum_{i\in V_{G}}L_{i}\left(0\right)}{N_{G}}, \end{array}$$

where β is the tolerance parameter for overload. According to Equation (2), each sensor has the same capacity, which is β times the average load of the initial network. Obviously, β relates to the cost of network construction. The larger β is, the more extra capacity resources each sensor owns to protect the network against cascading overloaded failures, but the higher the cost of constructing the network. So, a trade-off should be made regarding the robustness of the network and its construction cost. Undoubtedly, by introducing appropriate β, we can guarantee that there are no overloaded sensors in the initial network. However, if the load of a sensor at time *t* exceeds its capacity, it will malfunction at time t+1.

### 2.3. Load Redistribution Schemes

If a sensor malfunctions, the load traffic should be rerouted to bypass it toward the destinations. This redistribution of load is a transient action and may be global or local, depending on the routing policies. In fact, global and local routing policies both exist [19]. For this consideration, the following two redistribution schemes of load are proposed to imitate the multisink-oriented redistribution process of load in commonly used routing schemes.

#### 2.3.1. Global Routing (GR)

For the GR scheme, each sensor can acquire the real-time topological information of the entire network to make the shortest path routing decision. In this case, if a sensor malfunctions, its load will be reassigned globally within the network. Thus, it is natural to assume that once a sensor *i* fails at time t, it is removed from the network and the whole network topology is updated. A sensor’s load in the network is then renewed according to Equation (1).

**Figure 1:**
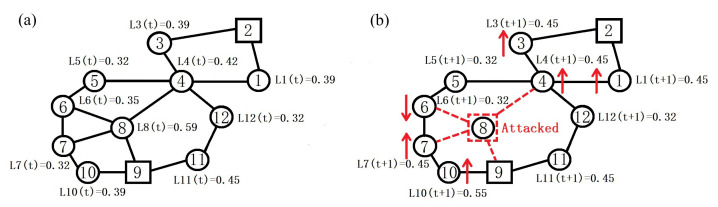
An example of redistribution of load under the GR scheme. (**a**) Initial load distribution. (**b**) Redistribution of load after sensor 8 is attacked.

Figure 1 gives an example of redistribution of load under the GR scheme. In this example, nodes 2 and 9 are sinks, and the rest are sensors. Suppose that sensor 8 is attacked and removed from the network. In comparison with the network load distribution before the attack (Figure 1a), the load of more than half of the sensors in the network changes after the attack (Figure 1b). Among them, the load of sensors 1, 3, 4, 7 and 10 increases. Before sensor 8 is attacked, the data at sensors 5 and 6 reach sink 9 through sensor 8. When sensor 8 is attacked and fails, the data at sensor 5 have to go through sensor 4 to sensor 1 or 3 and finally to sink 2. Meanwhile, the data at sensor 6 need to reach sink 9 through sensors 7 and 10, causing an immediate increase of the load of these five sensors.

#### 2.3.2. Local Routing (LR)

For the LR scheme, each sensor can only acquire the real-time depth information of its neighbor nodes. In this case, if a sensor malfunctions, its original load will be redistributed to the neighbor nodes closest to a sink. Based on this, the following settings can be made for the LR scheme. Suppose that a sensor *i* fails and is removed from the network at time *t*; then, the load of its neighbor sensors at time t+1 is renewed according to

(3) $$\begin{array}{c} L_{j}\left(t+1\right)=\left\{\begin{array}{c} L_{j}\left(t\right)+\frac{L_{i}\left(t\right)}{|\Gamma_{i}\left(t+1\right)|},j\in \Gamma_{i}\left(t+1\right)\\ L_{j}\left(t\right),\;\mathrm{otherwise},\; \end{array}\right. \end{array}$$

where $\Gamma_{i}\left(t+1\right)$ is the node collection consisting of the sensors closest to a sink among the neighbor sensors of sensor *i* at time t+1. $|\Gamma_{i}\left(t+1\right)|$ is the size of the collection $\Gamma_{i}\left(t+1\right)$.

**Figure 2:**
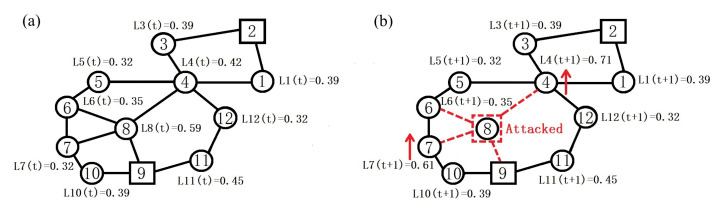
An example of redistribution of load under the LR scheme. (**a**) Initial load distribution. (**b**) Redistribution of load after sensor 8 is attacked.

Figure 2 illustrates an example of redistribution of load under the LR scheme in the same topology as Figure 1. Suppose that sensor 8 fails due to an attack. Among its neighbors, sensors 4 and 7 only take two hops to arrive at their respective nearest sink. Therefore, the load of sensor 8 is assigned equally to sensors 4 and 7, thus increasing the load of these two sensors.

### 2.4. Cascading Mechanism

Suppose that the potential cascading failure in the considered WSN is triggered by an initial attack on a single sensor. After removing this faulty node, the topology or connectivity of the network is altered, which causes the global or local redistribution of load according to the selected routing rule. When a sensor receives extra load, its updated load may exceed the capacity, and overloaded failure occurs consequently. In addition, the removal of the faulty node may make the network disintegrate into some subnetworks. For a subnetwork, if there are no sinks, then all sensors cannot communicate with any sink. All these sensors in the subnetwork are treated as failed nodes due to isolation, although they are not overloaded. Any failure causes a new redistribution of load and, as a result, subsequent failures may take place. This cascading process repeats until there are no more overloaded and isolated nodes.

### 2.5. Robustness Measure

The size of the giant connected component after cascade-based attacks is a widely used measure of network robustness [31,32,33]. This measure is reasonable for peer-to-peer networks that do not distinguish the nodes’ functions in the process of traffic transmission. However, in actual WSNs, when an attack occurs, the user is more concerned about the number of sensors that can still keep communication with at least one sink. We call this quantity the effective component size and consider it to examine the consequences of cascades on the network. Supposed the effective component size to be $R_{i}$ after the cascades triggered by a sensor *i*. To measure the robustness of the entire network against cascading failures, since $0\leq R_{i}\leq N_{G}-1$, we adopt the normalized effective component size, i.e.,

(4) $$\begin{array}{c} R\left(G\right)=\frac{\sum_{i\in V_{G}}R_{i}}{N_{G}\left(N_{G}-1\right)}, \end{array}$$

where the summation over all the effective component sizes is obtained by removing each sensor initially. A larger R(G) indicates that the network is more robust against cascading failures.

## 3. Impacts of Topological Parameters for Multisink Placement on Robustness

In order to investigate how multisink placement affects the robustness of WSNs against cascading failures, we consider five topological parameters for quantifying the sinks’ locations, namely the average degree of sinks, the average betweenness of sinks, the average efficiency of sinks, the average closeness of sinks, and the average shortest path length of sinks. For a connected network, the specific definitions of these five parameters are as follows.

(1) The average degree of sinks (ADS)
  (5) $$\begin{array}{c} ADS=\frac{1}{N_{S}}\sum_{i\in V_{S}}k_{i}, \end{array}$$
  where $k_{i}$ represents the degree of node *i*.
(2) The average betweenness of sinks (ABS)
  (6) $$\begin{array}{c} ABS=\frac{1}{N_{S}}\sum_{i\in V_{S}}B_{i}, \end{array}$$
  where $B_{i}$ denotes the betweenness [34] of node *i*.
(3) The average efficiency of sinks (AES)
  (7) $$\begin{array}{c} AES=\frac{2}{N_{S}\left(N_{S}-1\right)}\sum_{i\in V_{S}}\sum_{j\in V}d_{ij}, \end{array}$$
  where $d_{ij}$ represents the shortest path between nodes *i* and *j*.
(4) The average closeness of sinks (ACS)
  (8) $$\begin{array}{c} ACS=\frac{2}{N_{S}\left(N_{S}-1\right)}\sum_{i\in V_{S}}\frac{1}{\sum_{j\in V_{S},j>i}d_{ij}}. \end{array}$$
(5) The average shortest path length of sinks (ASPLS)
  (9) $$\begin{array}{c} ASPLS=\frac{2}{N_{S}\left(N_{S}-1\right)}\sum_{i\in V_{S},j\in V_{S},j>i}d_{ij}. \end{array}$$

Among them, the first two parameters, i.e., ADS and ABS, measure the centrality of positions of sinks in a network, while the last three parameters, i.e., AES, ACS and ASPLS, characterize the distribution of sinks, which can quantitatively evaluate the average ability of sensors to arrive at a sink within a smaller distance. Therefore, these quantities can represent the topological location properties of sinks from the physical and functional perspectives. Their modifications can initiate varying the multisink placement of the network that accordingly affects the network robustness against cascading failures.

Since random property and scale-free property are widely observed in real-life WSNs, it is natural and important to adopt a random network (RN) and scale-free network (SFN) to present an overall evaluation of how the above topological parameters for multisink placement affect network robustness. We apply the proposed cascading model considering different routing schemes (i.e., GR and LR) on these two typical networks. Network science tells us that the degree distribution of RN topology is homogeneous, while the degree distribution of SFN topology is heterogeneous. Note that “network topology” here defines how all nodes within a network are connected to each other no matter whether it is a sensor or a sink. Clearly, there exist four network scenarios for WSNs, which can be illustrated by the combinations of network structure and routing scheme, including RN-GR, SFN-GR, RN-LR and SFN-LR.

Our simulations are based on Matlab. To imitate real-life network conditions, the RN and SFN we adopted are generated referencing the topology construction algorithms [10]. IEEE 802.11b is used as the MAC layer, and the node’s transmission radius is set to 40 m. Regarding the wireless propagation, the Log Normal Shadowing Model (LNSM) [35] has been used to obtain the distance between the nodes based on the measurements of the Received Signal Strength Indication (RSSI) [36]. To make a fair comparison, for each network topology, the total number of nodes is fixed as N=150, and the average degree is set to 4. Based on the network topology, a fixed number of $N_{S}=6$ sinks will be deployed, and other nodes are sensors. Moreover, due to the limitation of network construction cost, the capacity of every sensor is generally not very high, and we set the tolerance parameter to β=1. In this work, our main concern is the cascading process under varying conditions of topological locations of sinks for WSNs, and it does not involve the impact of the MAC layer on the network. In addition, we do not take into account the impact of energy factors (e.g., residual energy) because the cascading process is much faster than the energy-depletion failure process caused by load transmission.

**Figure 3:**
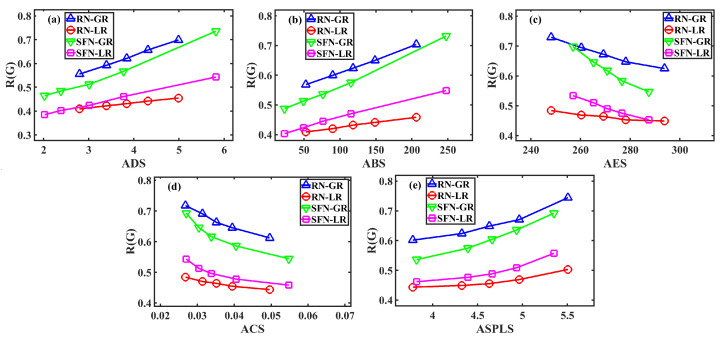
Impact of different topological parameters on network robustness. (**a**) The average degree of sinks (ADS). (**b**) The average betweenness of sinks (ABS). (**c**) The average efficiency of sinks (AES). (**d**) The average closeness of sinks (ACS). (**e**) The average shortest path length of sinks (ASPLS).

For each network scenario, to discuss the impact of multisink placement on the cascading robustness of WSNs, the sinks’ locations are randomly assigned 1000 times. For each distribution in each network, we can obtain a set of values of topological parameters (i.e., ADS, ABS, AES, ACS and ASPLS) and the resulting robustness R(G) of the network under this case. Thus, a total of 1000 pairs of values for each topological parameter and R(G) can be obtained. Next, we sort these 1000 pairs of data according to the corresponding topological parameter values and then divide them into 5 groups: specifically, the first 200 pairs of data form group 1, the following 200 pairs of data form group 2, and so on. For each group, we compute the average of 200 topological parameter values and the average of 200 R(G) values, respectively. In this way, for each network scenario, we can obtain five pairs of values, which give a five-point curve in Figure 3.

From each subgraph in Figure 3, we can see four different curves, indicating that the robustness is different under different network structures and routing schemes. Compared with the network structure, the robustness difference of varying routing schemes is more pronounced. We can easily observe that for networks of any structure (RN or SFN), the curve of the GR is obviously above that of the LR, which means that the GR is more conducive to network robustness. This is because compared with the LR, during the load redistribution process of the GR, more network nodes can share the load of the failed nodes, reducing the load increment of these load-sharing nodes, thus inhibiting their overload failures and the possible failure propagation.

More importantly, it is clear that the topological parameters describing the sinks’ locations exert important impacts on the robustness. For all curves of each subgraph in Figure 3, with the increase of ADS, ABS or ASPLS, the performance of robustness increases. Conversely, increasing AES or ACS reduces the robustness.

In order to understand these results, we can categorize the five topological parameters into three groups. First, ADS and ABS are quantities that measure the centrality of positions of sinks in a network. Sinks can be considered to be deployed as hubs, which are at central positions with larger ADS and ABS. If the sinks are located with higher centrality, it is advantageous for sensors to obtain better access to the sinks. In this case, the lower the network load, the harder it is for sensors to overload and be removed from the network. The more difficult it is also for the sensors to be disconnected from the sinks and cause isolated failures. These inhibit cascading propagation, which makes the network more robust. AES is a quantity that directly evaluates the mean distance between sinks and sensors. A smaller AES means that a sensor is more easily accessible to a nearby sink, making the network load lower and more balanced and resulting in higher network robustness. The final ACS and ASPLS give the relative location among sinks. With a smaller ACS or a larger ASPLS, sinks are distributed in a more decentralized way, which is favored for the robustness of WSNs.

It should be noted that in Figure 3, the considered WSNs have quite different network structures or/and quite different routing schemes. As expected, the network structure and routing scheme both affect the evolution of the curves for the WSNs. We can observe that given any network structure (or routing scheme), varying the sinks’ locations, the change in the robustness of WSNs with GR (or SFN topology) appears to be more obvious than that with LR (or RN topology). However, the evolution trend of the curves for these WSNs is the same, as shown in each subgraph of Figure 3. It suggests the universality of our finding that a WSN in which its sinks are distributed in a decentralized manner and connect as many sensors as possible has better robustness against cascading failures.

## 4. Optimal Multisink Placement

A better robust WSN against cascading failures has a greater capacity to sustain its normal and efficient functioning. To obtain the optimal multisink placement making the network as robust as possible and identify topological properties behind this optimal placement, in this part, we formulate an optimization problem for maximizing the robustness R(G) when the sinks’ locations can be modified.

In a complex WSN, this optimization is an NP-hard problem. Here, we employ a nature-inspired algorithm called simulated annealing (SA) [37] to find the optimal solution of this problem, which can be described as follows.

(1) Randomly select $N_{S}$ nodes as sinks in the initial network $G_{0}$, and then compute the robustness $R(G_{0})$. Set the time step t=1.
(2) Randomly select a sink and modify its location randomly to obtain the new network $G_{t}$. Then, compute $R(G_{t})$.
(3) $G_{t}$ is accepted with the probability
  (10) $$\begin{array}{c} p=\left\{\begin{array}{c} 1,R\left(G_{t}\right)\geq R\left(G_{t-1}\right)\\ e^{(R\left(G_{t}\right)-R\left(G_{t-1}\right))/T},otherwise \end{array}\right., \end{array}$$
  where *T* denotes the temperature parameter and is a function of the time step *t*.
(4) If the value of $R(G_{t})$ remains unchanged in a large number of the latest consecutive time steps (set as 2000), the algorithm is stopped. Otherwise, set t=t+1 and then go to step 2.

During the implementation of the algorithm, the temperature parameter *T* should be progressively decreased from an initial value that is large enough. This enables the search of solutions to escape from the local optimum and ensures that the system can finally reach an equilibrium. In our study, the cooling schedule of the temperature *T* follows the exponential rule $T\left(t\right)=T_{0}\theta^{t}$, where $T_{0}$ is the initial temperature and θ is a cooling coefficient. Here, we set these two parameters following the rules in Chapter 15 of [38].

The proposed SA algorithm is applied to the same network scenarios as the previous section. The results of topological parameters and robustness before and after optimization are shown in Table 1. For all four scenarios, compared to the initial network, the robustness of the optimized network is significantly improved. Specifically, with the GR, the robustness of the initial RN and SFN is 0.654 and 0.610, respectively, while the optimal robustness is increased by 0.212 and 0.234, respectively. With the LR, the robustness of the original RN and SFN is 0.435 and 0.462, respectively, while the optimal robustness is improved by 0.162 and 0.192, respectively. The results show the effectiveness of our algorithm. By comparing these improvement results, one can see that given any network structure (or routing scheme), the increase in the robustness of the network with GR (or SFN topology) appears to be more obvious than that with LR (or RN topology). This is consistent with the result in the previous section. In this sense, we can say that the robustness of a WSN with the GR is more sensitive to its sinks’ locations than that with the LR, especially when the heterogeneous SFN topology is selected as the network structure.

Moreover, for different network scenarios, when comparing the topological parameters before and after optimization, ADS, ABS and ASPLS are all increased after the optimizations, while AES and ACS are both decreased. These results are what we found in the previous section. Hence, it can be concluded that the robustness of a WSN with any structure and routing scheme against cascaded attacks can be effectively enhanced by allocating sinks as hubs (making ADS and ABS larger) and decentralizing these sinks in the network (making AES and ACS smaller and ASPLS larger). Considering this issue while designing WSNs will make them more robust against cascading failures.

## 5. Conclusions

In this paper, based on an actual cascading model, a WSN is investigated in terms of its multisink placement, and the association with the cascading robustness is established. The proposed cascading model takes into account the multisink-oriented load distribution characteristics and the two most commonly used routing schemes for WSNs. The statistical parameters considered for quantifying the sinks’ locations include ADS, ABS, AES, ACS, and ASPLS. Then, the impact of varying these quantities on network robustness are discussed in detail using the random network and the scale-free network imitating real-life network conditions. Our experimental results reveal that in order to enhance the robustness of a WSN against cascading failures, the *ADS*, *ABS* and *ASPLS* should be larger, and the *AES* and *ACS* should be smaller, which means that its sinks should be arranged as hubs, and these sinks should be uniformly distributed in the network. These results are effective in different network structures and routing schemes studied, and they are also verified through our designed simulated annealing algorithm optimizing multisink placement. Moreover, both the network structure and routing scheme can affect the sensitivity of the robustness of the network to its sinks’ locations.

Our study clearly illustrates the importance of considering multisink placement when optimizing the robustness of WSNs to resist cascading failures. In reality, how to engineer a large-scale WSN that has more cascading robustness is crucial in many applications. Our findings provide an efficient and easy way to do this: just manipulate the topological locations of a small number of its sinks in a reasonable manner. The self-organizing characteristic of WSNs which have a topology that can be flexibly tuned according to actual needs [39,40] also provides favorable conditions for the implementation of this solution.

In the present work, aiming to explore the impact of varying the topological location properties of sinks on network cascading robustness for WSNs, we mainly focus on the modeling of a multisink-oriented load-transfer process where the two routing schemes considered are topology-aware only. In the next step, we will extend the model by considering more practical routing factors (e.g., the congestion extent of sensor nodes and residual energy). On this basis, we will figure out efficient ways to comprehensively analyze the influence of topological properties (e.g., clustering, average path length) of both the connectivity of the network and the distribution of sinks on the cascading process and launch a more robust network design for real-life multisink WSNs. The interesting challenge of this work is to make sure that the topology structure designed is robust to cascading failures and can also meet other network performance requirements, such as delivery latency and energy efficiency.

## Figures and Tables

**Table 1:** Topological parameters and robustness in different scenarios before and after optimization where the sinks’ locations can be modified. The results are the average of 50 independent runs.

Scenarios	Optimization	*ADS*	*ABS*	*AES*	*ACS*	*ASPLS*	*R*(*G*)
RN-GR	before	3.83	110.56	278.13	0.040	4.33	0.654
	after	5.67	281.42	220.93	0.031	5.93	0.866
RN-LR	before	3.83	110.56	278.13	0.040	4.33	0.435
	after	5.50	253.45	221.27	0.032	5.87	0.597
SFN-GR	before	3.67	88.42	274.67	0.042	4.20	0.610
	after	7.33	348.48	236.53	0.032	5.80	0.844
SFN-LR	before	3.67	88.42	274.67	0.042	4.20	0.462
	after	6.83	328.72	240.53	0.033	5.67	0.654

## Data Availability

The data presented in this study are available in this article.
